# Emotional Responses and Self-Protective Behavior Within Days of the COVID-19 Outbreak: The Promoting Role of Information Credibility

**DOI:** 10.3389/fpsyg.2020.01846

**Published:** 2020-07-31

**Authors:** Žan Lep, Katarina Babnik, Kaja Hacin Beyazoglu

**Affiliations:** Department of Psychology, Faculty of Arts, University of Ljubljana, Ljubljana, Slovenia

**Keywords:** COVID-19, mass communication, information credibility, negative emotions, self-protective behaviors, psychological response, health communication

## Abstract

Due to changes in the information environment since the last global epidemic, high WHO officials have spoken about the need to fight not only the current COVID-19 pandemic but also the related infodemic. We thus explored how people search for information, how they perceive its credibility, and how all this relates to their engagement in self-protective behaviors in the crucial period right after the onset of COVID-19 epidemic. The online questionnaire was circulated within 48 h after the first case of COVID-19 was confirmed in Slovenia. We gathered information on participants’ demographics, perception of the situation, their emotional and behavioral responses to the situation (i.e., self-protective behavior), perceived subjective knowledge, perceived credibility of different sources of information, and their level of trust. We looked into the relationships between perceived credibility and trust, and self-protective behavior of 1,718 participants and found that mass media, social media, and officials received relatively low levels of trust. Conversely, medical professionals and scientists were deemed the most credible. The perceived credibility of received information was linked not only with lower levels of negative emotional responses but also with higher adherence to much needed self-protective measures, which aim to contain the spread of the disease. While results might vary between societies with different levels of trust in relevant governmental and professional institutions, and while variances in self-protective behavior scores explained by our model are modest, even a small increase in self-protective behavior could go a long way in viral epidemics like the one we are facing today.

## Introduction

With the emergence of social networks and their omnipresence, especially as a source of information in critical situations, the information environment has become significantly more complex since the last worldwide epidemic of H1N1 influenza. Today, people are faced with an abundance of information from various sources, many of them not credible, and the way key information is relayed to the public has become critical (The Lancet, 2020). As result, high-ranking officials from the World Health Organization (WHO) have recently spoken about the need to fight not only the current COVID-19 pandemic but also the related infodemic. In the present research, we were thus interested in how different informational outlets (besides media, we also analyzed the communication of various officials) can shape perceptions, emotional responses, and whether credible communication can promote behavioral responses (i.e., adherence to preventive and protective measures) to the novel crisis situation.

Indeed, extant research shows that the perceived quantity and credibility of information received correlates with adherence to infection prevention behavior, e.g., frequent hand washing, avoiding close contact, etc (Etingen et al., 2013), which is crucial in fighting the spread of the disease. While media can help with promoting healthy behavior change (Sandman, 2009), this is exceedingly important in the early stages of the epidemic (Xiao et al., 2015), when the possibility of its containment is highest. Alarmist framing and intensive reporting of mass media can, on the contrary, spark fear and even hysteria (Van den Bulck and Custers, 2009), resulting in the reduced possibility of mobilizing the public (Sherlaw and Raude, 2013). Such emotions can be further amplified by prolonged exposure to negative reporting, while personal experience with the disease is limited (Brug et al., 2004; Lau et al., 2011).

The level of trust in sources of information also plays an important role in motivating the engagement in self-protective behaviors. However, results may depend on the source of information – a higher level of trust in official government communication was found to result in higher self-efficacy and hand washing. Conversely, relying on informal interpersonal information results in heightened perceived threat and avoidance behaviors (Liao et al., 2010). Additionally, the perceived credibility of various sources of information also varies greatly in the eyes of the public. For example, King et al. (2018) found that parents exhibited high levels of trust in doctors, and less so in the government during the H1N1 outbreak. In accordance with this, research on the H1N1 epidemic has shown that people were doubtful about recommendations made by the government (Teasdale and Yardley, 2011).

As government recommendations are a special form of health care communication, they are subject to harsh evaluation in terms of credibility, feasibility, and costs (Teasdale and Yardley, 2011). Nonetheless, it is crucial for people to follow those recommendations in case of a health threat. At the same time, in cases when information relayed by public health officials is deemed less credible, people can turn to online news, interpersonal networks and social media for information regarding an outbreak (Jang and Baek, 2019). These latter sources can be less trustworthy and filled with inaccurate information. Moreover, research has found that people who have consulted with their doctor are more likely to adopt self-protective behavior (Lin et al., 2018), supporting the notion that doctors have a special role in communicating information regarding self-protective behavior. Based on previous studies (e.g., King et al., 2018) on the trust in and perceived credibility of different institutions at the time of epidemics, our first hypothesis was that perceived trust and credibility would be highest for medical doctors, scientists, and medical institutions. At the same time, we expected that the trust and perceived credibility of politicians and political institutions would be lower, as previous research has shown that people tend to trust them less (King et al., 2018) and that epidemics can have a further negative impact on these perceptions (Bangerter et al., 2012; Yeung et al., 2017).

The cases of countries where COVID-19 spread rapidly are telling in how important it is for people to know (and apply) basic protective measures in order to contain the spread of the disease, especially in the critical period after the first few confirmed cases, when the possibility of containment is the highest. Hence, it seems important that crucial information about the current pandemic is communicated by credible sources – for example, health care professionals and scientists (e.g., epidemiologists, virologists). In the present study, we were thus interested in how initial perceptions and responses were formed within hours of the first confirmed case of COVID-19 in Slovenia. We also wanted to know how these responses related to the perceived trust and credibility of information sources available to the population.

The first case of COVID-19 in Slovenia was confirmed relatively late, on March 4 2020, after the disease had already spread to all neighboring countries, most notably Italy. However, the media had been covering the global spread of the disease extensively since January of that year (e.g., the online media outlet with highest reach in Slovenia published the first major article on January 9th). We thus looked into how people gathered information about the COVID-19 outbreak, how they rated the credibility of different informational outlets at the time, and what their emotional response to the threat was. Previous studies have shown that people’s anxiety tends to increase sharply at the beginning of an epidemic (Cheng and Cheung, 2005). For this reason, we expected that general feelings of concern and fear of contracting COVID-19 would increase significantly after the first confirmed case of COVID-19 in Slovenia. At the same time, we were interested in the size of the change, seeing as the time span between our two points in time was only between 2 and 4 days.

Furthermore, as Slovenia was largely unaffected by previous epidemics such as SARS and H1N1, this could have resulted in lowered public awareness and knowledge about dealing with and containing the spread of infectious diseases. Thus, we were also interested in how informed people felt about the epidemic and self-protective measures and how the perceived credibility of information sources is linked to emotional responses, knowledge of self-protective behaviors, and adherence to them. Specifically, we were interested in who crucial information should be relayed by in order to boost self-protective behavior and support the effort of officials and medical professionals to contain the spread of the virus. As mentioned above, previous studies have shown that higher trust and perceived credibility are positively associated with self-protective behavior (Liao et al., 2010; Etingen et al., 2013). At the same time, it was found that trust in government and medical institutions helped reduce anxiety and that negative emotions are associated with self-protective behavior (Cheung and Tse, 2008). In addition, studies found relationships between people’s trust in institutions and their subjective knowledge of the disease at the time of the epidemic (Freimuth et al., 2014), alongside some evidence of a link between subjective knowledge and self-protective behavior. However, this link did not appear to be consistent (Leung et al., 2005).

In literature, there is no consensus on the relationship between trust and credibility in the realm of health-related information seeking (Sbaffi and Rowley, 2017). Though correlation has been proposed, the distinctions made in the literature are unclear. In the present research, we thus focused on both. We specifically stressed the perceived credibility of information sources related to the COVID-19 pandemic, as well as the general trust in institutions that were involved in spreading information about COVID-19 in Slovenia and abroad. We proposed and tested two structural models linking each of the constructs to self-protective behavior, which was mediated by the effect of negative emotions and subjective knowledge about the disease.

## Materials and Methods

### Participants

In total, 4,000 people have responded to the survey. Of those, 2,424 gave their informed consent and 1,722 completed the survey (81.7% were women, eight stated their gender as other and were excluded from gender-differences analyses). Before data analysis, four participants were disregarded due to their age (under 18), which prevented them from providing valid consent. The analysis was therefore performed on a sample of 1,718 participants. The average age of 1,718 participants was between 18 and 81 (*M* = 37.95, *SD* = 13.76); they varied in terms of their education and resided in all statistical regions of Slovenia (most lived in the central region, which is also the most populated), but were slightly younger and more educated than Slovenian population (SiStat, 2020a, b). See Supplementary Table 1 for a detailed demographic description of the sample.

### Study Design and Procedure

Data collection for this cross-sectional study started within 48 h of the first confirmed COVID-19 case in Slovenia. The survey was hosted on a local survey hosting platform 1ka.si that complies with national and European General Data Protection Regulation, guaranteeing participants’ anonymity.

The survey was posted on the department’s social media accounts and targeted residents of Slovenia over the age of 18 (the age of consent). In addition, the link was shared on forums and circulated through the institute’s and researchers’ own mailing lists using a snowball sampling method (the survey was shared by more than 80 individuals).

As the goal of the study was to capture the public’s first impressions of the outbreak, the data were collected over the weekend on March 7th and 8th – still within the first 100 h after the first case. The time window was necessary to ensure the homogeneity of data, while minimizing the influence of concurrent developments. For example, the Slovenian National Security Council was called in session on March 9th, and two of the three public universities in Slovenia suspended their operation, thereby justifying the adequacy of our decision.

### Measures

The measures presented were a part of a battery of tests. We assessed the participants’ perceptions of the situation, their emotional and behavioral responses to the situation (i.e., self-protective behavior), their perceived subjective knowledge and trust, as well as the perceived credibility of different sources of information (all measures are presented in the Supplementary Material). We also collected demographic information (age, gender, educational level, and region of residence). Additionally, we assessed the participants’ objective knowledge about COVID-19 by utilizing information available on the official website of the National Institute of Public Health (NIPH). However, seeing as during this period official information was rapidly changing, we decided to omit the scores from further analyses, as it was unclear which of the answers were “correct” at the time of responding.

The selection of measures was guided by our research questions and based on both the measures used in previous epidemics, as well as the review of fast-report articles on the COVID-19 epidemic that were available at the time of planning the study. All measures were translated and, when needed, adapted to the Slovenian context of the COVID-19 epidemic using the standard forward-back translation method (two Slovenian native speakers performed independent translations from English to Slovenian; back translation to English was conducted by an expert in the language). As no psychometric data were available for used measures, we conducted a series of analyses to test their validity (see “Statistical Methods”).

Emotional responses to the situation were assessed using 11 items, with participants indicating their agreement on a five-point scale. The items, relevant to the viral outbreak, were selected and adapted from various psychological tools for assessing anxiety (e.g., *Following the information about the coronavirus outbreak makes me feel nervous.*; Beck et al., 1988; Spitzer et al., 2006) and rumination (e.g., *Because of what is happening in connection to the coronavirus outbreak, I find it hard to concentrate on my work.*; Nolen-Hoeksema and Morrow, 1991).

Additionally, five items were included to assess the perception of the COVID-19 outbreak (e.g., *How do you rate the severity of COVID-19 disease today?, How did you rate the severity of COVID-19 disease before the coronavirus appeared in Slovenia?*). They measured the degree of concern and fear of contracting the disease, perceived severity, perceived possibilities of containing the spread of COVID-19, and the amount of thinking about the disease using a six-point scale corresponding to the question (e.g., *1* – *not severe at all, 6* – *very severe*). Those items were adapted from Li et al. (2020). As the study had a cross-sectional design, participants were asked to assess their perceptions retrospectively (before the first confirmed case of COVID-19 in Slovenia), and their current perceptions (after the first confirmed case).

Participants’ perceived subjective knowledge about COVID-19, about symptoms and about self-protective behaviors was measured using three items. Participants indicated their agreement on a five-point scale (e.g., *I think I know the symptoms and the course of the COVID-19 disease*). Actual engagement in self-protective behavior was assessed using 10 items with a three-point scale (*does not apply to me*, *partly applies to me*, and *totally applies to me*). Different self-protective behaviors (e.g., *more frequent hand washing*, *less frequent touching of one’s face*, *avoiding crowds*, etc.) were identified using guidelines posted on the NIPH and WHO websites. We also included some behaviors that are not efficient in preventing the spread of the virus (e.g., *buying a supply of food or health supplies*), but were identified by NIPH as frequent among the population.

Following previous studies on health-related self-protective behavior in the realm of vaccination (Gust et al., 2005; Jolley and Douglas, 2014), we assessed the participants’ overall trust in different institutions (*How would you rate your trust in the following people and institutions in general, unrelated to the reporting on coronavirus: politics, Ministry of Health, NIPH, the health care system, general practitioners, scientists, mass media and social media*). We also assessed the perceived credibility of information about the COVID-19 outbreak received from different spokespersons in the media (*Please rate how credible you find the information about the coronavirus that you received in the media from: Ministry of Health representatives, NIPH representatives, Medical chamber representatives, medical doctors, scientists, journalists*). Both were assessed using a five-point scale. Participants were also asked where they gather information about COVID-19 (*TV, radio, newspapers, online news portals, social media*).

### Statistical Methods

First, data were screened for missing variables – between 0 and 1.9% of data was missing with observed variables. In analyses where groups were compared or single-item measures were used, the participants with missing data were excluded. When computing scales, missing values were imputed with the item medians. In confirmatory factor analyses and structural equation modeling, case-wise maximum likelihood estimation was applied. Data were analyzed in R using psych (Revelle, 2019), WRS2 (Mair and Wilcox, 2019), and lavaan (Rosseel, 2012) packages. The reliabilities of the scales were assessed using Revelle’s omega and in scales, comprising two items, using Spearman–Brown coefficient which is more appropriate for such cases (Ayearst and Bagby, 2010; Eisinga et al., 2012).

As items assessing emotional responses had been drawn from various measures, we ran an exploratory factor analysis (EFA), using half of the sample (*n* = 853) to explore the homogeneity of collected data. Since various solutions were not clear, we retained only one factor, which explained 40% of the variance, and included items measuring degrees of nervousness, concern, feelings of hopelessness, and problems with concentration. The factor was then tested using confirmatory factor analysis (CFA) with the second half of the sample (*n* = 854), which supported the proposed solution [MLR estimator, χ^2^(2) = 15.83, *p* < 0.001, CFI = 0.98, TLI = 0.94, RMSEA = 0.09 (95% CI = 0.05–0.13), SRMR = 0.03; see Supplementary Table 2 for factor loadings]. The resulting scale had sufficient reliability (ω = 0.73).

Using the same procedure, a three-factor solution for self-protective behaviors was supported: personal hygiene (frequent hand washing, not touching one’s face, ρ = 0.55), social contacts (avoiding close contact, avoiding any contact, not leaving one’s home, not attending mass events, not traveling, ω = 0.91), and the preparatory behaviors factor (stocking on food and supplies, stocking on health supplies, ρ = 0.47). See Supplementary Table 3 for factor loadings.

To test whether the perceived trust in different representatives and institutions and perceived credibility of information relayed by different spokespersons was affected by an underlying perceptual clustering (e.g., reporting less trust in all institutions perceived as political), we again ran – in each case – EFA followed by CFA. Based on parallel analysis, the EFA (minimum residual factoring method and oblimin rotation) for the perceived trust suggested the retention of three factors, explaining 59% of the variance in scores. The three factors are public institutions (politics in general, the Ministry of Health, the National Institute of Public Health, ω = 0.87), professionals (doctors, the healthcare system as a whole, scientists, ω = 0.78), and media (traditional mass media and social media, ρ = 0.50). Subsequent CFA exhibited adequate fit [MLR; χ^2^(16) = 88.96, *p* < 0.001, CFI = 0.97, TLI = 0.95, RMSEA = 0.08 (95% CI = 0.06–0.10), SRMR = 0.04, see also Supplementary Table 4].

EFA for the perceived credibility of information – ran with the same specifications as above – suggested a two-factor solution. The perception of the credibility of information sources might be explained by two factors – perceived as professionals (doctors, scientists, and medical chamber representatives, ω = 0.85) and perceived as non-professionals/officials (journalists, representatives of Ministry of Health, representatives of NIPH, ω = 0.83) – these account for 66% of the variance in scores. Again, CFA supported the proposed solution [MLR; χ^2^(7) = 40.54, *p* < 0.001, CFI = 0.98, TLI = 0.96, RMSEA = 0.09 (95% CI = 0.07–0.12), SRMR = 0.04, see also Supplementary Table 5].

### Ethics Approval Statement

All procedures performed in studies that involved human participants were in accordance with the ethical standards of the institutional research committee (Ethics Commission of the Faculty of Arts, University of Ljubljana, no. 181-2020) and with the 1964 Helsinki declaration and its later amendments or comparable ethical standards.

## Results

The participants reported some degree of concern and fear of contracting COVID-19 even before the first Slovenian case of COVID-19 was confirmed. The reported severity of the disease and perceived possibilities of containing its spread before it reached Slovenia were rated at about the midpoint (see Table 1), with females being slightly more afraid and perceiving the disease as more severe.

**TABLE 1:** Mean scores and gender differences for perceptions of different aspects of COVID-19 before and after the first confirmed case with changes in perceptions after the first confirmed case in Slovenia.

	**Before the first case in Slovenia**				**After the first case in Slovenia**				**Change after the first case**	
	**M_male_ (SD)**	**M_female_ (SD)**	***W***	***p***	**M_male_ (SD)**	**M_female_ (SD)**	***W***	***p***	*t*(*df*)*	***d***
Worrying	2.25 (1.36)	2.32 (1.35)	199,050	0.26	3.01 (1.52)	3.36 (1.50)	178,200	< 0.001	−31.35 (1014)	0.47
Severity	3.10 (1.46)	3.31 (1.39)	186,540	0.01	3.42 (1.45)	3.77 (1.38)	178,760	< 0.001	−16.95 (1014)	0.22
Containing	3.23 (1.59)	3.22 (1.50)	206,460	0.97	3.06 (1.63)	3.04 (1.59)	205,200	0.89	4.91 (1012)	0.08
Fear of contracting	2.07 (1.31)	2.31 (1.38)	184,540	0.01	2.67 (1.52)	3.22 (1.60)	164,790	< 0.001	−26.82 (1013)	0.40
Thinking	2.94 (1.48)	2.92 (1.44)	207,640	0.90	3.85 (1.55)	4.03 (1.51)	192,450	0.07	−31.53 (1015)	0.49

Due to non-normal distribution of the variables, Mann–Whitney test was used for calculating gender differences and Yuan–Welch test for repeated measures was used for calculating changes after the first confirmed case. All items were rated on a five-point scale. * All ps < 0.001.

Within 2 days of the first confirmed Slovenian case of COVID-19, however, our participants reported a significant change in all the assessed perceptions. During this period, they were more concerned and afraid, thinking more about the disease, perceived it as more severe, and rated the chances of its containment as worse, regardless of gender (see Table 1). However, gender differences emerged in the extent of the change – females reported, on average, more than one whole point of change in concern (see Table 1). The presence of negative emotions was also higher in females (*M* = 2.71, *SD* = 0.97) than males [*M* = 2.54, *SD* = 0.95; *t*(447, 03) = -2.84, *p* = 0.005]. At the same time, females reported higher subjective knowledge of self-protective behavior and exhibited more self-protective behavior than males (see Table 2).

**TABLE 2:** Mean scores and gender differences for subjective knowledge about self-protective behaviors and and engagement in self-protective behaviors.

	**M_male_ (*SD*)**	**M_female_ (*SD*)**	***W***	***p***
Subjective knowledge	4.08 (1.01)	4.25 (0.91)	189,650	0.01
Personal hygiene	2.08 (0.64)	2.28 (0.60)	172,400	< 0.001
Social contacts	1.65 (0.59)	1.79 (0.58)	178,400	< 0.001
Preparing	1.32 (0.54)	1.43 (0.57)	185,330	< 0.001

**Items were rated on a three-point scale (1, not at all; 2, partly; 3, completely); due to non-normal distribution of the variables, Mann-Whitney test was used.**

In terms of gathering information about COVID-19, most participants used online news portals as their source (74.1%), followed by television news (65.7%) and social media (61.0%). Around half (55.3%) of the participants used the official webpage of the NIPH, where all official information is gathered in the style of WHO. Lastly, radio and health care professionals were the source of information for a minority of the participants (27.7 and 11.0%, respectively).

Unrelated to the current COVID-19 outbreak, people generally trusted scientists the most, followed by their general practitioners. The health care system and NIPH received midline scores, while politics and social media were rated the lowest (see Table 3). Similarly, participants viewed the information they received about COVID-19 from scientists and doctors as most credible, while information relayed by journalists, representatives of the Ministry of Health (MoH) and Medical chamber was perceived as less credible (see Table 3). There were no significant gender differences in perceived credibility, and only small differences in trust in politics (*W* = 225,660, *p* = 0.02) and scientists (*W* = 225,220, *p* = 0.02); in both cases, males reported a slightly higher level of trust (0.18 and 0.12 points, respectively). Moreover, we observed a notable correlation between general trust and the perceived credibility of information received from MoH (*r* = 0.71, *p* < 0.001), NIPH (*r* = 0.80, *p* < 0.001), and medical doctors (*r* = 0.54, *p* < 0.001). Further correlation was found between trust in the health care system and the perceived credibility of medical chamber representatives (*r* = 0.58, *p* < 0.001) and medical doctors (*r* = 0.57, *p* < 0.001), between trust in science and the perceived credibility of scientists (*r* = 0.67, *p* < 0.001), and finally between trust in mass media and the perceived credibility of journalists in reporting about COVID-19 (*r* = 0.63, *p* < 0.001).

**TABLE 3:** Overall trust in various institutions and perceived credibility of information recieved by various sources.

	***N***	***M***	***SD***	**Mdn**	**Skew**	**Kurt**	**SE**
**Trust**							
Politics	1,701	1.96	1.06	2	0.91	0.12	0.03
Ministry of Health	1,703	2.75	1.18	3	0.07	–0.83	0.03
NIPH	1,702	3.15	1.29	3	–0.25	–0.97	0.03
Health care system	1,698	3.00	1.15	3	–0.16	–0.71	0.03
GPs	1,699	3.60	1.15	4	–0.57	–0.40	0.03
Scientists	1,697	3.81	1.07	4	–0.85	0.34	0.03
Mass media	1,698	2.38	1.01	2	0.30	–0.40	0.02
Social media	1,697	1.99	0.90	2	0.66	0.11	0.02
**Credibility**							
Journalists	1,694	2.81	1.01	3	–0.03	–0.34	0.02
MoH representatives	1,693	3.16	1.10	3	–0.17	–0.59	0.03
NIPH representatives	1,693	3.43	1.18	4	–0.41	–0.65	0.03
Medical chamber representatives	1,689	3.18	1.11	3	–0.20	–0.55	0.03
Medical doctors	1,687	3.52	1.04	4	–0.43	–0.22	0.03
Scientists	1,691	3.86	1.00	4	–0.77	0.30	0.02

**FIGURE 1:**
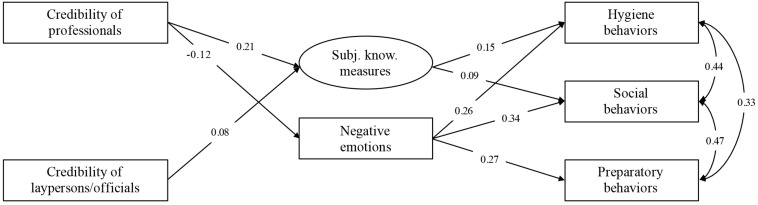
Model for predicting the adherence to self-protective behaviors from perceived credibility of informational sources via subjective knowledge of proposed measures and negative emotions [χ^2^(9) = 49.94, *p* < 0.001, CFI = 0.97, TLI = 0.93, RMSEA = 0.06 (95% CI = 0.04–0.07), SRMR = 0.03, BIC = 17080.69; MLR estimator]; all paths significant at *p* < 0.05.

Two structural models were tested using R package lavaan (Rosseel, 2012) to explore how trust and perceived credibility of news sources are linked to self-protective behavior (see Figure 1 for the model, containing credibility, and Supplementary Figure 1 for the model containing trust scores). The model with credibility scores exhibited good fit to the data. It suggested that the perceived credibility of news relayed by medical professionals and scientists is linked to lower negative emotions and higher subjective knowledge of self-protective behaviors. Subjective knowledge is in turn linked to higher engagement in self-protective behaviors, and so is the experience of negative emotions. The model explained roughly 9% of personal hygiene, 8% of preparatory, and 12% of social contact behavior (see Figure 1).

## Discussion

One of our research interests was the impact of the first confirmed case of COVID-19 in Slovenia on the general level of concern and fear of contracting the virus. We expected that the general feeling of concern and fear of contracting COVID-19 would be consistent with previous research (e.g., Cheng and Cheung, 2005) and increase significantly after the first confirmed case. In line with this, our results have shown that within 2 days of the first confirmed case, participants reported a significant change in all perceptions assessed. The participants reported that they were now more worried and anxious, thought more about the disease, perceived it as more severe and assessed the chances of containing the disease as worse than before the first confirmed Slovenian case of COVID-19. As this was a cross-sectional study, perceptions for the time before the first Slovenian case of COVID-19 were reported retrospectively. This may have lowered the accuracy of reporting, as participants, influenced by their concurrent emotional state, may have given biased reports. However, the length between our points in time was short (between 2 and 4 days), so the extent of the possible bias is likely not great.

Most participants searched for information online, either through mass or social media. At the same time, they exhibited relatively low levels of trust in either of these sources. The same goes for politicians and public institutions. This is consistent with our hypothesis that the trust in and perceived credibility of politicians and political institutions will be lower, as previous research has shown that people tend to trust them less (King et al., 2018). In addition, studies from various countries have shown that this trust tends to decrease even further in times of epidemics (Bangerter et al., 2012; Yeung et al., 2017). Interestingly, NIPH was clustered perceptually by the participants to the same factor as politics and exhibited relatively low levels of trust and perceived credibility. This is especially problematic, as measures they propose could be perceived as less useful, feasible, and worth following (Teasdale and Yardley, 2011), while they should in theory be the most credible and professionally sound. Conversely, information provided by medical professionals and scientists was rated as most credible, suggesting that crucial information should perhaps be communicated by them.

This is especially important in the early phases of the outbreak, when medical staff is not yet preoccupied with caring for people who have contracted the disease, and when self-protective measures are most efficient (Xiao et al., 2015; Lin et al., 2018). Moreover, our results support previous findings (Etingen et al., 2013) about the relation between perceived credibility and behavioral actions. In summary, future policy change should include the optimization of communication channels by emphasizing the role of professionals in communication. Of special importance is online communication, where people gather most information about the spread of the disease and learn of various protective measures. Specifically, in the case of NIPH, by designing a professional body, the officials should underline their professional credentials when appearing in media, increase their presence on social media, and include the voice of medical practitioners in their press releases. In later stages, however, the relaying of information could be passed to scientists relevant to the situation.

Previous research (e.g., Cheung and Tse, 2008; Liao et al., 2010; Etingen et al., 2013; Freimuth et al., 2014) has shown significant relationships between perceived credibility and trust, emotions, subjective knowledge, and self-protective behavior. In our study, we proposed and tested two structural models that explain the role of perceived credibility of information sources and perceived trust in various institutions that are engaged in the communication related to the epidemic. The model that exhibited better fit was the proposed model, which linked the perceived credibility of information sources to engagement in self-protective behaviors via negative emotions and perceived knowledge of self-protective measures. This suggests that information relayed by credible sources can lead to lower levels of negative emotional responses, which can be important as epidemics are emotionally taxing. Even though the variances in self-protective behavior scores explained by our model are modest, even a small boost in engagement in self-protective behaviors could go a long way in viral epidemics like the one we are faced with today and help lower the number of infected people (aka flattening of the curve).

The structural model, which included trust in various institutions, exhibited worse fit. The reason for this could partly be that trust was assessed in general, whereas credibility of information sources was assessed directly in connection to the epidemic situation. Even though trust and credibility in our study were strongly linked with each other, the difference between assessing them could be the reason for the lower predictive value of the model including trust.

The research was conducted in the early stages of the epidemic. At that point, emotional responses might not have been as severe as in the later stages. Additionally, the perceived importance of adhering to protective measures, along with the intensity of reporting on said measures, could be much lower, which could result in lower correlations. However, as the study was only conducted in Slovenia, it should be perceived as a case study. The results may not be easily transferable to other societies, especially those where governments receive high levels of trust or use different means of informing the public. In future studies, our findings should thus be cross-culturally validated, and explored in later stages of the epidemic.

Furthermore, our study has some limitations in terms of sampling – the sample was slightly younger than the average age of the Slovenian population (43.5 years; SiStat, 2020b), and the percentage of people with tertiary education was higher than the national average (SiStat, 2020a). This, coupled with the fact that data were collected online, could mean that the sample is biased in terms of information literacy and stated sources of information. The study could also not reach some of the most vulnerable groups in the current epidemic (e.g., the elderly). However, during the epidemic, other means of data collection are less feasible, and specific groups likely differ from the general population in terms of their perceptions, responses, but also needs (e.g., stricter protective measures). While females were also overrepresented in the sample, they were similar to males in terms of demographics, and no differences were observed in perceived trust and credibility of information sources. As the context of the study deviated significantly from the everyday, measures used were not validated beforehand, which could cause concern in terms of validity. While the reliabilities of used scales were adequate (Ayearst and Bagby, 2010), especially the scales comprising two items could be expanded with additional items in order to ensure higher confidence in obtained scores. Besides cross-cultural validation and comparisons of perceived credibility of informational sources, and key officials in pandemic situations, more detailed qualitative or mixed-methods studies would also contribute to better understanding of collective perceptions and adherence to self-protective measures.

Effective health communication – or even communication that is perceived by people to be effective in terms of credibility – remains crucial in adopting protective measures and fighting misinformation. This conclusion has several potential implications for health communication practice. In early stages of communication, medical professionals and scientists have a higher credibility potential, suggesting they should be intensively included in public communication and disseminate important health-related information and advice on proper protective measures. Moreover, our results suggest that such communication could be effective in positively reframing the pandemic situation. It would serve as a protective factor in an emotionally taxing environment, where isolation measures have left people without interpersonal contact, uncertain and afraid as to what the future might hold for them, in terms of both health and their financial status.

## Data Availability Statement

The raw data supporting the conclusions of this article will be made available by the authors, without undue reservation, to any qualified researcher.

## Ethics Statement

The studies involving human participants were reviewed and approved by the Ethics Commission of the Faculty of Arts, University of Ljubljana (No. 181-2020). Written informed consent for participation was not required for this study in accordance with the national legislation and the institutional requirements.

## Author Contributions

KH and KB conducted the literature review. ŽL conducted the analyses and interpreted the data. ŽL and KH drafted the manuscript. KB provided the critical feedback. All authors contributed to study design, data collection, and read the final version of the manuscript and approved it for publication.

## Conflict of Interest

The authors declare that the research was conducted in the absence of any commercial or financial relationships that could be construed as a potential conflict of interest.
