# Supplementary material for: Emotional Responses and Self-Protective Behavior Within Days of the COVID-19 Outbreak: The Promoting Role of Information Credibility
Source: Front Psychol. 2020 Jul 31;11:1846. doi: 10.3389/fpsyg.2020.01846 (PMC7411328; doi:10.3389/fpsyg.2020.01846)
Supplement: Supplementary file 1 [file Table_1.pdf]

## Supplementary Material

### 1 Demographic data

Table 1. *Demographic characteristics of the sample*

| | male | | female | | total[1] | |
|---|---|---|---|---|---|---|
| | *n* | % | *n* | % | *n* | % |
| gender | 302 | 17.83 | 1384 | 81.70 | 1694 | |
| | | | | | | |
| age | 301 | | 1371 | | 1686 | |
| 18-20 | 34 | 11.30 | 88 | 6.42 | 124 | 7.35 |
| 21-30 | 90 | 29.90 | 406 | 29.61 | 499 | 29.60 |
| 31-40 | 68 | 22.59 | 329 | 24.00 | 403 | 23.90 |
| 41-50 | 61 | 20.27 | 269 | 19.62 | 332 | 19.69 |
| 51-60 | 31 | 10.30 | 166 | 12.11 | 197 | 11.68 |
| 60+ | 17 | 5.65 | 113 | 8.24 | 131 | 7.77 |
| | | | | | | |
| occupational status | 302 | | 1379 | | 1696 | |
| student | 73 | 24.17 | 280 | 20.30 | 356 | 20.99 |
| working | 191 | 63.25 | 871 | 63.16 | 1071 | 63.15 |
| uneployed | 12 | 3.97 | 77 | 5.58 | 90 | 5.31 |
| retired | 16 | 5.30 | 118 | 8.56 | 135 | 7.96 |
| other | 10 | 3.31 | 33 | 2.39 | 44 | 2.59 |
| | | | | | | |
| education | 301 | | 1367 | | 1683 | |
| primary | 9 | 2.99 | 24 | 1.76 | 34 | 2.02 |
| secondary | 110 | 36.54 | 443 | 32.41 | 558 | 33.16 |
| tertiary | 153 | 50.83 | 770 | 56.33 | 932 | 55.38 |
| doctorate | 29 | 9.63 | 130 | 9.51 | 159 | 9.45 |
| | | | | | | |
| region | 299 | | 1365 | | 1678 | |
| Mura | 8 | 2.68 | 46 | 3.37 | 54 | 3.22 |
| Drava | 26 | 8.70 | 139 | 10.18 | 166 | 9.89 |
| Carinthia | 3 | 1.00 | 35 | 2.56 | 38 | 2.26 |
| Savinja | 16 | 5.35 | 109 | 7.99 | 127 | 7.57 |
| Central Sava | 8 | 2.68 | 44 | 3.22 | 52 | 3.10 |
| Lower Sava | 4 | 1.34 | 32 | 2.34 | 36 | 2.15 |
| Southeast Slovenia | 18 | 6.02 | 64 | 4.69 | 83 | 4.95 |
| Littoral-Lower Carniola | 13 | 4.35 | 54 | 3.96 | 67 | 3.99 |
| Central Slovenia | 145 | 48.49 | 600 | 43.96 | 753 | 44.87 |
| Upper Carniola | 26 | 8.70 | 92 | 6.74 | 118 | 7.03 |
| Gorizia | 15 | 5.02 | 61 | 4.47 | 77 | 4.59 |
| Coastal-Karst | 17 | 5.69 | 89 | 6.52 | 107 | 6.38 |

*Note*: [1]totals differ due to missingnes and reported gender being “other”.

Table 1. *Demographic characteristics of the sample (continued)*

| | male | | female | | total[1] | |
|---|---|---|---|---|---|---|
| | *n* | % | *n* | % | *n* | % |
| chronic health problems | 297 | | 1356 | | 1668 | |
| yes | 47 | 15.82 | 301 | 22.20 | 353 | 21.16 |
| no | 250 | 84.18 | 1055 | 77.80 | 1315 | 78.84 |
| had COVID-19 like symptoms | 297 | | 1370 | | 1684 | |
| yes | 18 | 6.06 | 70 | 5.11 | 90 | 5.34 |
| no | 279 | 93.94 | 1300 | 94.89 | 1594 | 94.66 |
| knows someone infected | 300 | | 1380 | | 1695 | |
| yes | 9 | 3.00 | 49 | 3.55 | 60 | 3.54 |
| no | 291 | 97.00 | 1331 | 96.45 | 1635 | 96.46 |

*Note*: [1]totals differ due to missingnes and reported gender being “other”.

## 2 Measures

Please note that not all items presented here were used in our calculations. See *Statistical methods* section in the manuscript for details.

### 2.1 Emotional responses

Please rate to what extent you agree with the following statements. *(1 – completely disagree, 5 – completely agree)*

1. Following the news about the coronavirus outbreak makes me feel nervous.
2. Obtaining news about the coronavirus outbreak calms me.
3. These days, every conversation turns to the coronavirus.
4. I feel overwhelmed with the news about the coronavirus.
5. If I don’t obtain new information about the coronavirus in the news sources I use regularly, I find additional sources.
6. If I can, I try to distance myself from the news about the coronavirus.
7. I stopped following the news about the coronavirus.
8. The news about the coronavirus bore me.
9. The news about the coronavirus burden me.
10. I feel helpless about the coronavirus situation.
11. Because of what is happening in connection to the coronavirus outbreak, I find it hard to concentrate on my work

### 2.2 Perceptions of the COVID-19 outbreak

*1 - not severe at all, 5 - very severe*

1. How worried are you about the COVID-19 disease today?

2. How worried were you about COVID-19 disease before the coronavirus appeared in Slovenia?
3. How do you rate the severity of COVID-19 disease today?
4. How did you rate the severity of COVID-19 disease before the coronavirus appeared in Slovenia?
5. How do you rate the possibility of containing the spread of the COVID-19 disease today?
6. How did you rate the possibility of containing the spread of the COVID-19 disease before the coronavirus appeared in Slovenia?
7. How afraid are you of the COVID-19 disease today?
8. How afraid were you of the COVID-19 disease before the coronavirus appeared in Slovenia?
9. How often do you think about the COVID-19 disease today?
10. How often were you thinking about the COVID-19 disease before the coronavirus appeared in Slovenia?

### 2.3 Subjective knowledge

Please rate your agreement with the following statements. *(1 – completely disagree, 6 – completely agree)*

1. I think I have all the information about (the spread of) the COVID-19.
2. I think I know the symptoms and the course of the COVID-19 disease.
3. I think I know the protective measures against the spread of the COVID-19 outbreak.

### 2.4 Overall trust in the institutions and people

How would you rate your trust in the following people and institutions in general, unrelated to the reporting on coronavirus? *(1 – I distrust completely, 5 – I trust completely)*

1. Politics in general
2. Ministry of Health
3. National Institute of Public Health
4. The health care system
5. General practitioners
6. Scientists
7. Mass media (e.g., television, radio, newspapers)
8. Social media

### 2.5 Gathering of information about the COVID-19 outbreak

Where do you gather the information about (the spread of) the COVID-19? Please check all that apply.

1. Television
2. Radio
3. Newspapers and magazines
4. Online news portals
5. Social media
6. The official web-sites of the public institutions (e.g., website of the National Institute of Public Health)
7. Directly from the medical staff

## 2.6 Perceived credibility of information about the COVID-19 outbreak

Please rate how credible you find the information about the coronavirus that you received in the media from. *(1 – not at all credible, 5 – completely credible)*

1. Ministry of Health representatives
2. National Institute of Public Health representatives
3. Medical chamber representatives
4. Medical doctors
5. Scientists
6. Journalists

## 2.7 Self-protective behavior

Please rate whether the statements bellow apply to you. *(does not apply to me, partly applies to me, totally applies to me)*

1. I wash my hands more frequently.
2. I touch my face less frequently.
3. I am avoiding close contact with other people (e.g., handshakes).
4. I am avoiding any, even distant contact with other people.
5. I am avoiding crowded places and events.
6. I spend more time at home.
7. I follow the news more frequently.
8. I have cancelled an international trip/travel.
9. I stocked up on essential supplies (e.g., food)
10. I stocked up on medical or health supplies (e.g., face masks, disinfectants).

# 3 Confirmatory factor analyses

Table 2. *Factor loadings for negative emotion scale*

| Item | estimate | *SE* | *p* | standardized factor loading |
|---|---|---|---|---|
| nervousness | 0.91 | 0.05 | <0.001 | 0.67 |
| concern | 0.94 | 0.05 | <0.001 | 0.70 |
| helplessness | 0.76 | 0.05 | <0.001 | 0.57 |
| having trouble concentrating | 0.69 | 0.05 | <0.001 | 0.59 |

*Notes*. $\chi^2_{(2)} = 15.83$, $p < 0.001$, CFI = 0.98, TLI = 0.94, RMSEA = 0.09 (95% *CI* = 0.05–0.13), SRMR = 0.03 (MLR estimator)

Table 3. *Factor loadings for protective behaviors factors*

| Factor/item | estimate | *SE* | *p* | standardized factor loading |
|---|---|---|---|---|
| Personal hygiene | | | | |
| hand washing | 0.49 | 0.02 | <0.001 | 0.70 |
| not touching face | 0.55 | 0.02 | <0.001 | 0.79 |
| Social contacts | | | | |
| avoiding close contact | 0.52 | 0.02 | <0.001 | 0.77 |
| not attending mass events | 0.63 | 0.02 | <0.001 | 0.77 |
| staying home | 0.57 | 0.02 | <0.001 | 0.74 |
| not travelling | 0.46 | 0.02 | <0.001 | 0.52 |
| avoiding any contact | 0.58 | 0.02 | <0.001 | 0.80 |
| Preparatory behavior | | | | |
| food and supplies | 0.42 | 0.02 | <0.001 | 0.69 |
| hygiene items | 0.50 | 0.02 | <0.001 | 0.71 |

*Notes.* $\chi^2_{(24)}$ = 145.98, $p$ < 0.001, CFI = 0.97, TLI = 0.95, RMSEA = 0.06 (95% *CI* = 0.05–0.07), SRMR = 0.03 (WLSMV estimator)

Table 4. *Factor loadings for trust factors*

| Facor/item | estimate | *SE* | *p* | standardized factor loading |
|---|---|---|---|---|
| Political institutions | | | | |
| politics at large | 0.56 | 0.04 | <0.001 | 0.52 |
| MoH | 1.06 | 0.03 | <0.001 | 0.89 |
| NIPH | 1.23 | 0.03 | <0.001 | 0.93 |
| Professionals | | | | |
| healthcare system at large | 1.07 | 0.03 | <0.001 | 0.92 |
| medical doctors | 0.76 | 0.04 | <0.001 | 0.66 |
| scientist | 0.66 | 0.04 | <0.001 | 0.60 |
| Media | | | | |
| mass media | 1.89 | 0.60 | 0.001 | 1.87 |
| social media | 0.24 | 0.09 | 0.006 | 0.26 |

*Notes.* $\chi^2_{(16)}$ = 88.96, $p$ < 0.001, CFI = 0.97, TLI = 0.95, RMSEA = 0.08 (95% *CI* = 0.06–0.10), SRMR = 0.04 (MLR estimator); set covariation between politics at large and MoH ($r$ = 0.38)

Table 5. *Factor loadings for credibility factors*

| Factor/item | estimate | *SE* | *p* | standardized factor loading |
|---|---|---|---|---|
| Laypersons/officials | | | | |
| journalists | 0.47 | 0.04 | <0.001 | 0.47 |
| MoH representatives | 1.04 | 0.03 | <0.001 | 0.93 |
| NIPH representatives | 1.09 | 0.03 | <0.001 | 0.92 |
| Professionals | | | | |
| medical doctors | 0.82 | 0.04 | <0.001 | 0.78 |
| scientists | 0.63 | 0.04 | <0.001 | 0.63 |
| Medical chamber representatives | 0.96 | 0.03 | <0.001 | 0.85 |

*Notes.* $\chi^2_{(7)} = 40.54$, $p < 0.001$, CFI = 0.98, TLI = 0.96, RMSEA = 0.09 (95% *CI* = 0.07–0.12), SRMR = 0.04 (MLR estimator); set covariation between medical doctors and scientists ($r = 0.38$)

## 4 Supplementary Figure

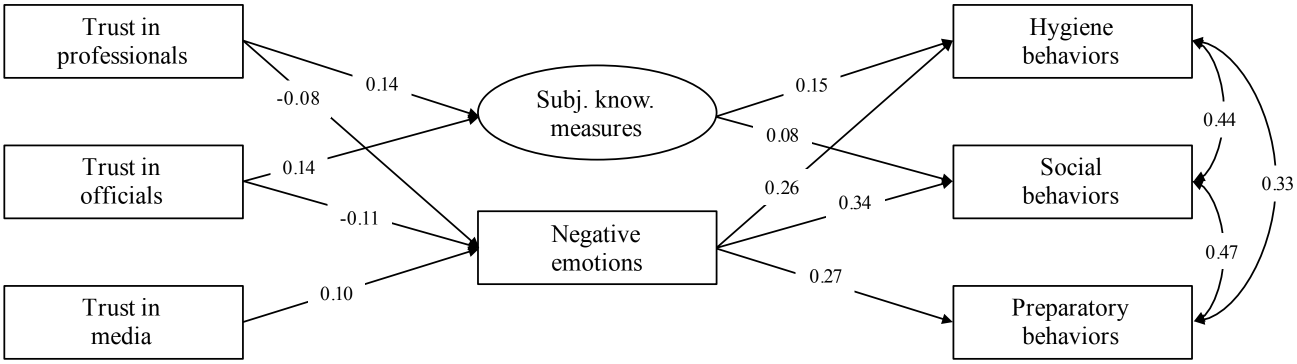


**Supplementary Figure 1.** Model for predicting the adherence to self-protective behaviors from trust via subjective knowledge of proposed measures and negative emotions ($\chi^2_{(12)} = 168.23$, $p < 0.001$, CFI = 0.90, TLI = 0.78, RMSEA = 0.09 (95% *CI* = 0.08–0.10), SRMR = 0.05, BIC = 17090.81; MLR estimator); all paths significant at p < 0.05
